# Benefit from the Chin-Down Maneuver in the Swallowing Performance and Self-Perception of Parkinson's Disease Patients

**DOI:** 10.1155/2017/7460343

**Published:** 2017-01-19

**Authors:** Annelise Ayres, Geraldo Pereira Jotz, Carlos R. M. Rieder, Maira Rozenfeld Olchik

**Affiliations:** ^1^Postgraduate Program in Health Sciences, Universidade Federal de Ciências da Saúde de Porto Alegre, Porto Alegre, RS, Brazil; ^2^Postgraduate Program in Medical Sciences, Universidade Federal do Rio Grande do Sul, Porto Alegre, RS, Brazil; ^3^Department of Neurology and Postgraduate Program in Rehabilitation, Universidade Federal de Ciências da Saúde de Porto Alegre (UFCSPA), Porto Alegre, RS, Brazil; ^4^Department of Surgery and Orthopaedics, Universidade Federal do Rio Grande do Sul, Porto Alegre, RS, Brazil

## Abstract

*Aims*. To verify the effectiveness of the maneuver application in swallowing therapy with PD.* Materials and Method*. We performed an open-label trial, with three groups compounds by PD individuals: the experimental group, control group, and orientation group. The study included PD patients with dysphagia. A cognitive screening, through a questionnaire about depression and quality of life, was conducted. Swallowing assessment was performed through (1) fiberoptic endoscopic evaluation of swallowing (FEES); (2) clinical evaluation and Functional Oral Intake Scale (FOIS); and (3) assessment of the quality life related to swallowing (SWALQOL). A therapeutic program, which consisted of chin-down postural maneuver and orientations on feeding, was applied. Both groups (EG and OG) received on-month therapeutic program.* Results*. A significant improvement in swallowing, evaluated by clinical assessment, was observed in solid (*p* < 0.001) and liquid (*p* = 0.022) consistencies in EG when compared to OG and CG. Patients in EG presented improvement in QoL, with the significant difference in comparison with the other groups, about domain frequency of symptoms (*p* = 0.029) in SWALQOL questionnaire.* Conclusion*. The postural maneuver chin-down improved swallowing performance and self-perception, but not the laryngeal signs. This trial is registered with registration number NCT02973698.

## 1. Introduction

Dysphagia is a highly prevalent symptom in Parkinson's disease (PD) [1]. In the early stages of the disease, it may be present in up to 80% of patients and, in advanced stages, the incidence may increase to 95% [1–3]. Signs and symptoms of dysphagia in PD may occur in oral, pharyngeal, and esophageal swallowing phases. Laryngeal penetration and pulmonary aspiration are the most aggravating symptoms [4–6]. Such signs and symptoms may result in complications, such as malnutrition, dehydration and lung problems. Respiratory infection by aspiration is the leading direct cause of death in patients with PD, and it is highly associated with immobility and dysphagia [7]. There is prevalence from 30% to 45% of pneumonia among the death causes in patients with PD [8–11].

Although dysphagia is a complicated PD symptom, the number of studies about the effects of swallowing therapy in this disease is still limited. There are studies on the use of biofeedback for the therapeutic intervention [12–14], about the use of expiratory muscle strength training for swallowing [15], about the effects of surface electrical stimulation [16], and on myofunctional exercises for swallowing [17, 18]. However, these pieces of evidence are far from conclusive. The only studies on rehabilitation and management of oropharyngeal dysphagia in patients with PD still do not have conclusive results [4, 19].

In a literature review on swallowing therapy for PD, we found only nine studies on this subject [4]. From these, only two [20, 21] reported the effect of chin-down posture maneuver. Logemann et al. [20] conducted, so far, the largest study on compensatory approaches for oropharyngeal dysphagia in PD. The authors compared chin-down posture maneuver with changes in food consistency in the treatment of oropharyngeal dysphagia. The results showed that the postural maneuver was the less effective strategy in the prevention of liquids aspiration than changes in food consistency. The same was found in a study by Robbins et al. [21]. This study demonstrated profound differences of interventions to prevent pneumonia. However, the authors suggest that swallowing becomes safer as the food consistency changes (thickened liquids). Other articles show improvements in some swallowing parameters, after the investigated treatment strategies (change of food texture, tactile and thermal oral stimulation, myofunctional exercises, Lee Silverman Voice, exercises with biofeedback, and expiratory muscle training), but no significant improvements in swallowing global function.

As was previously described, up to this moment, studies which investigate the effectiveness of the chin-down postural maneuver in the rehabilitation of dysphagia PD patients have applied it only in an isolated moment, during the performance of a swallowing objective examination. It does not enable a therapeutic process to learn the maneuver correctly. They have tested it only in one food consistency (liquid consistency), or they have applied it to other therapeutic strategies, with no opportunity to verify its isolated effectiveness. So, this study has the purpose of verifying the efficiency of the maneuver application, in isolation, in a therapeutic program, as well as testing its effectiveness with different food consistencies.

## 2. Materials and Methods

This study is an open-label trial performed in PD patients with oropharyngeal dysphagia, from March 2014 to May 2015. Clinical trial is registered with ClinicalTrails.gov number NCT02973698.

### 2.1. Participants

Participants were PD patients recruited from a Parkinson's disease and Movement Disorders Clinic from Hospital de Clínicas de Porto Alegre (HCPA), a reference hospital, in Rio Grande do Sul, Brazil. The written free and informed consent to participate in the research was obtained from participants. This study was approved by the hospital central research ethics committee.

### 2.2. Inclusion and Exclusion Criteria

The included subjects were patients with the diagnosis of Parkinson's disease, according to the criteria from the United Kingdom Parkinson's Disease Society Brain Bank criteria for idiopathic PD [22] that have the diagnosis of oropharyngeal dysphagia. The diagnosis of oropharyngeal dysphagia was made by fiberoptic endoscopic evaluation of swallowing (FEES), according to the criteria from Santoro et al. [23]. PD staging was based on the Hoehn and Yahr Staging Scale (H&Y, Degree of Disability Scale) (Hoehn & Yahr, 1967), used to assess the severity of PD. As exclusion criteria, there were the following aspects: presenting language and hearing disorders, which could complicate the understanding of an intervention program, diagnosis of dementia, or other neurological illnesses.

### 2.3. Patient Evaluation

Cognitive screening which consists of Mini-Mental State Examination (MMSE) and Montreal Cognitive Assessment (MoCA) in all patients was performed before the intervention. The instruments Parkinson Disease Questionnaire-39 (PDQ-39), translated to Portuguese, and the Beck Depression Inventory (BDI) were also applied. These tests were used to verify the influence of cognitive aspects, depression, and quality of life in the therapeutic process.

To evaluate the intervention's effectiveness, an evaluation of swallowing was conducted in two moments (before and after intervention). Three kinds of assessments were performed: (1) fiberoptic endoscopic evaluation of swallowing (FEES); (2) clinical evaluation; and (3) assessment of the quality of life related to swallowing (SWALQOL).

*(i) Fiberoptic Endoscopic Evaluation of Swallowing (FEES)*. This objective examination of swallowing was performed according to the following protocol: First, the prior state of secretion in the nasopharyngeal structures, oropharynx, and laryngopharynx was tested. Next, the individual received liquid consistency offered through a syringe, 3 and 5 mL of water with edible blue food coloring. For the sticky consistency, 3 and 5 mL of thickened water were provided through a needle with edible blue food coloring. A half a glass of water and salt biscuits with real blue food coloring were offered to assess the solid consistency. No anesthetic was used for the examination. The images were later analyzed by an otolaryngologist physician, experienced in the dysphagia area. The presence of thickening on the posterior laryngeal wall, tremor in structures (base of tongue and vallecula), early escape (characterized by the presence of food in the hypopharynx or larynx before the swallowing reflex was triggered), vallecular stasis in glossoepiglottic folds and pyriform sinus (characterized by accumulation of food after the third swallowing on the mentioned structures), penetration (characterized by the presence of food in the laryngeal vestibule), tracheal aspiration (characterized by food intake in the region located below the vocal folds, in the subglottic region and in the trachea, at any time of swallowing), and cough reflex was observed. The alterations were classified as present or absent. The equipment used was the flexible nasopharyngoscope Maschida ENT-III, 3.2 mm, with Xerônio Storz light source.

*(ii) Clinical Evaluation and Functional Oral Intake Scale (FOIS)*. Clinical evaluation of swallowing was performed by a certified speech therapist, previously trained to apply the protocols. All the assessments were conducted by the same professional. This assessment had the purpose of checking signs and symptoms of oropharyngeal dysphagia. Solid food consistency (half portion of bread) and liquid (100 mL of water) evaluated by free demand were used. The analyzed signs and symptoms were based on the area literature [24], which are history of aspiration pneumonia; alert state; interaction attention/ability; awareness of the swallowing problem; awareness of secretion; ability to manipulate flows; postural control; fatigability; anatomy and oral, pharyngeal, and laryngeal physiology; orofacial tonus; oral apraxia; orofacial sensitivity; gag pharyngeal contraction; saliva swallowing; cough and hawk; swallowing apraxia; oral residue; delayed swallowing reflex; reduction in laryngeal elevation; wet voice; and multiple swallowing. A total of 21 signs and symptoms were evaluated as present or absent. At the end of the objective and clinical evaluation, the intake of food was scored according to Functional Oral Intake Scale (FOIS). This scale scores the level of oral food intake of patients at specific levels, from 0 (restricted to alternative food pathway) to 7 (total oral intake with no restrictions), with the aim of monitoring the patients' evolution during the therapeutic process. The valid and reliable instrument has a coefficient of interrater reliability from 0.98 to 0.99, with Kappa coefficient average values between 0.86 and 0.91, with appropriate consensual validity (Kendall 0,90 agreement) and criterion validity (based on the Mann Assessment test of Swallowing Ability) [25]. A translated and validated version for Brazilian Portuguese was used [26].

*(iii) The Quality of Life in Swallowing Disorders*. The questionnaire Quality of Life in Swallowing Disorders (SWAL-QOL) was applied, to verify the symptoms presented by the patients, as well as their influence on the quality of life. This instrument has Alpha Cronbach coefficient higher than 0.80, except in one domain. Thus, it presents excellent internal consistency and short-term reproducibility. It is a sensitive scale to differentiate oropharyngeal dysphagia degrees of severity. The version translated and validated for Brazilian Portuguese was used [27]. All questionnaires were applied in a waiting room. The questions and possible answers were read by the researcher for all patients. The questionnaires application was performed individually, for each patient.

### 2.4. Intervention

Individuals who agreed to participate in this study were allocated to one of the three interventions groups: (1) experimental group (patients performed the chin-down posture maneuver), (2) control group (PD individuals without any intervention), and (3) orientation group (PD participants that received swallowing orientations).

*(1) The Experimental Group (EG) (Chin-Down Posture Maneuver Group)*. Patients received an intervention program consisting of four weekly individual sessions of 30 minutes. In these sessions, the training of chin-down postural maneuver with saliva and water was performed. The participants were trained to perform the maneuver twice a day, swallowing saliva, and during meals, throughout the week, at home. The participants received a form, so they recorded the number of times they performed the maneuver at home. It allowed the control of adherence, the importance of adherence to treatment being reinforced at each session. Besides, the subjects received instructions regarding feeding as follows.

*Swallowing Orientations*

*Environment during Feeding Time*

- Do the meals in a quiet location.
- Keep off television, radio or any other device that can distract you.
- Keep your attention on the food.
- Do not talk during feeding.
- Avoid making meals at times when you are sleepy or tired.
- Try to make meals when in the ON period of medication.

*Posture during Feeding*

- Sit next to the table.
- Keep your torso upright and your head up.
- Avoid lying down right after the end of the meal.
- Put small portions of food in the mouth.
- Try to chew the food well and many times before swallowing.
- Swallow more than once, until you are sure there is no residue in the mouth.
- If you have the sensation of food “stuck in the throat” swallow more your saliva.

*Meal Time*

- Maximum time of the main meal (breakfast, lunch, and dinner) is 30 minutes.
- Do not eat in a hurry.

*Oral Hygiene*

- Do not forget to brush your tooth after meals.
- Remember to brush your tongue.
- If you use the dental prosthesis, it should be removed and cleaned with tooth brushing after every meal, and the tongue should be brushed.
- In patients that do not have teeth, washing the mouth should be done with a brushing of the tongue and the remaining with gauze soaked with water and mouthwash.

All the instructions, as well as the explanation about the maneuver, were submitted to the patients through a written document.

*(2) The Control Group (CG) (PD Individuals without Any Intervention)*. The participants of this group underwent evaluation of swallowing, and the same assessment was repeated after four weeks, without any intervention during that period.

*(3) Orientations Group (OG)*. The individuals participated in an intervention program which consisted of four individual sessions a week, with 30 minutes. In these sessions, the instructions about feeding were performed. The people received all the instructions on a written document (see Swallowing Orientations). In the sessions, doubts about the guidelines and treatment adherence were verified. In this group, the chin-down postural maneuver was not applied.

The chin-down postural maneuver is performed by requesting the patient to swallow lowering the head with the intention of touching the chin in the neck. This maneuver promotes the major protection of the airways by posteriorization of the base of the tongue, with an enlargement of the vallecular space and displacement of the epiglottis to a more protective position.

The EG and OG interventions were used by the same researcher, previously trained. After the end of the research, the same swallowing therapy performed to the GE was offered to the CG and OG individuals.

### 2.5. Statistical Analysis

Statistical analysis was performed through the Statistical Package for Social Sciences (SPSS), version 20.0. For the primary outcome analysis, the Shapiro-Wilk normality test, the Student's *t*-test (clinical evaluation of swallowing variable), and the Generalized Estimating Equations (GEE) (SWALQOL variable) were used. Data from the FEES variable did not allow inferential statistical analysis. The Spearman correlation test was used, with separate groups, to check the relationship between the demographic and clinical variables of the three groups and the clinical evaluation of the swallowing variable. The regression test and the Pearson correlation test were applied to check the influence of the BDI and the PDQ variables in the swallowing clinical evaluation. To verify the group's similarities in the preintervention assessment, the Shapiro-Wilk normality test was performed. Next, to the standard variables (age, MOCA, PDQ, and BDI), the ANOVA was applied. For the nonnormal variables (disease duration, education, and H&Y stages), the Kruskal-Wallis test for independent samples was used. For all tests, 5% of error was provided.

## 3. Results

**Figure 1:**
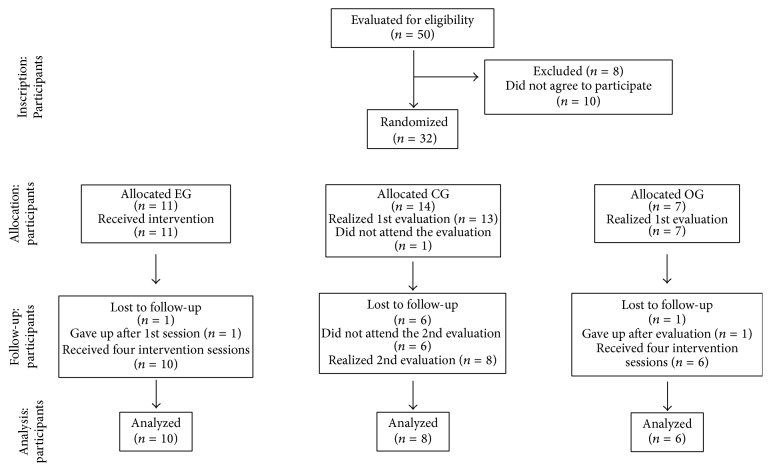
Steps of study.

The selection process of the participants consisted of 4 steps as it is shown in Figure 1. The sociodemographic and clinical profile of this study's participants are described in Table 1.

Patients that received the program of chin-down posture maneuver presented significant improvements in signals and symptoms of dysphagia by clinical evaluation when compared with the other two groups (control and orientation group) regarding solid (*p* < 0.001) and liquid (*p* = 0.022) consistencies (Table 2).

The analysis by fiberoptic endoscopic evaluation of swallowing (FEES) did not show differences between patients that received and did not receive the intervention.

Regarding quality of life, patients that received the intervention of chin-down maneuver (EG) presented a significant improvement in scores of domains frequency of symptoms and mental health on the SWAL-QOL when compared with the groups that did not receive this intervention (Table 3).

In the analysis of the correlation between the groups and cognitive variables with an evaluation of swallowing no correlations were found. Thus, no influence of the cognitive aspects of the therapeutic process was evidenced, in this population.

## 4. Discussion

Our results showed that patients that received the intervention of chin-down postural maneuver have improvement in clinical evaluation of dysphagia and quality of life evaluated by SWAL-QOL. These findings suggest that a simple and inexpensive dysphagia therapy as the chin-down postural maneuver is effective to improve dysphagia in PD patients. This intervention demonstrated benefits, even in a short period. Cognitive ability, depression, and quality of life did not influence this process.

In literature, there is heterogeneity studies about swallowing therapy in PD patients. At this moment, only eight studies about this topic in this population were found. These five studies [12, 14, 16–18] led to improvements in some participants' swallowing parameters, but with no global function changes. Three studies [13, 15, 28] found improvements in swallowing after therapy. These changes have been demonstrated through better scores in FOIS scale and Penetration-Aspiration Scale. Although our study had much less time of intervention, the results are similar to the literature [12, 14, 16–18]. Regarding SWALQOL, we observed that these results corroborated with other studies that found that swallowing-related QoL improved after an SLP intervention. This finding demonstrates the importance of dysphagia therapy in this population, as a way of reducing swallowing-related complaints and improving QoL [29].

In addition to the motor symptoms of PD, there is a high prevalence of nonmotor symptoms, such as cognitive decline, loss of facial expression, micrograph, and sleep disorders [30]. Therefore, clinical studies on swallowing therapy in PD need to control the influence of these symptoms in the therapeutic process. Among these symptoms, the cognitive decline is highlighted, which has a prevalence from 25% to 38.2% in early stages of PD [31, 32], and it may result in significant rehabilitation impacts. The lack of cognitive data associated with therapeutic improvements is critical to the right study evaluation, described in the literature [13, 15, 28].

Swallowing treatment in a neurodegenerative disease seeks functionality of safe swallowing and maintenance of nutritional status and respiratory conditions. According to the literature [20, 21], the choice of introducing a therapeutic strategy must follow the order: first, postural techniques; then, oral sensitivity improvements techniques; next, swallowing maneuvers; and, finally, change of food consistency. This law is based on the easiest way patients learn, and it aims at delaying the food texture change, which, in many cases, will not be reversed. Thus, the chin-down postural maneuver was chosen based on previous studies which have shown that it is among the two most recommended maneuvers in the therapeutic process for individuals with PD and based on its effectiveness to prevent aspiration pneumonia in patients with PD, when combined with liquid thickening [18, 20, 21, 28].

Also, this strategy aims to improve the protection of the airways, reducing the possibility of penetration in the larynx or aspiration, being frequently recommended for thin liquids swallowing, to reduce early escape and possible entry into the air pathway [18, 28]. Added to these aspects, according to a larger study [20] patients who received swallowing therapy in PD suggest that the chin-down posture is easier and more pleasant, compared to other used therapeutic strategies.

The therapeutic process involves changing habits and new learning. The postural maneuver performance involves learning the proper movement for its implementation, as well as inhibition of an automatic behavior for the implementation of a new way of swallowing. Thus, the individuals go through a learning process, which causes changes in structures and functioning of their neural cells and connections [33]. The consolidation of a new learning process depends on the plasticity of the central nervous system, which occurs in three stages: development, learning, and after lesional processes, translated into the nervous system attempting to form new connections and recovery of lost connections [33]. Thus, the lack of changes in the swallowing pattern, in an objective examination of the EG participants, may be a result of the short therapy time, which did not allow the complete knowledge consolidation.

The findings generalization is limited because the changes occur only from the clinical judgment. Although significant changes in the objective evaluation of swallowing have been observed, the results expressed impacts on participants' swallowing performance and self-perception, as evidenced by improvements in quality of life (SWALQOL) in the experimental group.

Also, the absence of changes in the FEES evaluated parameters must be analyzed with caution. It raises the hypothesis that this is a short intervention period for improvements in these parameters, and the FEES evaluates a unique feeding moment, with small food quantities and in an unusual manner to the patient. On the other hand, the clinical evaluation performed according to the demand allows a swallowing evaluation in an environmental situation. Besides, a high number of patients felt uncomfortable when they performed the FEES, which may negatively influence the findings.

Furthermore, the small sample is a limiting aspect of the findings, and it may have affected the results. However, these results may support larger and randomized studies to ensure the effectiveness of the chin-down postural maneuver for swallowing dynamics, quality of life, and swallowing difficulties in PD.

## 5. Conclusions

In conclusion, the present study shows that the chin-down posture maneuver, a free and easy procedure, improves the swallowing performance and self-perception of PD patients.

## Figures and Tables

**Table 1:** Sociodemographic and clinical data.

Groups	Experimental (EG)	Control (CG)	Orientation (OG)	*p* value
	*n* = 10	*n* = 8	*n* = 6	
Male gender	80%	75%	66.70%	—
Age^*∗*^	62.0 (±11.5)	62.8 (±6.2)	64.5 (±5.6)	0.694^a^
Schooling^*∗*^	5.9 (±4.1)	10.3 (±8.4)	12.0 (±9.1)	0.142^b^
Time of disease^*∗*^	10.7 (±4.7)	11.8 (±8.0)	8.8 (±6.0)	0.524^b^
H&Y^*∗*^	2.8 (±0.8)	2.5 (±0.7)	2.5 (±0.8)	0.363^b^
MEEM^*∗*^	26.6 (±3.8)	25.6 (±3.7)	25.3 (±4.1)	0.702^b^
MOCA^*∗*^	21.9 (±4.9)	20.5 (±7.7)	21.2 (±8.4)	0.834^a^
PDQ-39^*∗*^	41.4 (±13.8)	38.7 (±16.7)	36.5 (±17.1)	0.704^a^
BDI^*∗*^	13.8 (±7.7)	17.1 (±9.2)	14.7 (±9.3)	0.523^a^
FOIS^*∗*^	5.9 (±1.3)	6.8 (±0.5)	6.8 (±0.4)	—

^a^ANOVA; ^b^ Kruskal-Wallis test; ^*∗*^Representation of the data as mean and standard deviation; H&Y: Hoehn & Yahr Scale; MMSE: Mini-Mental State Examination; MOCA: Montreal Cognitive Assessment; PDQ-39: Parkinson's Disease Questionnaire-39; BDI: Beck Depression Inventory; FOIS: Functional Oral Intake Scale. — indicates that statistical testing was not applied to this variable (FOIS).

**Table 2:** Correlation between the periods before and after clinical evaluation of swallowing.

Groups	Solid			Liquid		
	Pre	Post	*p*	Pre	Post	*p*
	Mean (PD)	Mean (PD)		Mean (PD)	Mean (PD)	
Experimental (EG)	4.56 (±0.631)	2.40 (±0.290)	0.002^*∗*^	3.33 (±0.609)	1.56 (±0.388)	0.01^*∗*^
Control (CG)	4.88 (±1.007)	3.00 (±0.354)	0.083	2.13 (±0.874)	1.13 (±0.482)	0.26
Orientation (OG)	2.67 (±0.304)	3.17 (±0.495)	0.33	0.50 (±0.312)	0.83 (±0.281)	0.39

Student's *t*-test; PD: standard deviation; *p*: *p* value; ^*∗*^*p* < 0.005.

**Table 3:** SWALQOLL analysis before and after intervention.

Domain		Experimental (EG)		Control (CG)		Orientation (OG)		*p* value
		Mean (standard deviation)						
(1) Swallowing as a burden	Pre	71.5	9.4	98.2	6.8	89.3	8.0	0.279
	Post	60.4	13.8	85.7	9.9	95.6	8.4	
(2) Desire to eat	Pre	68.2	7.0	91.2	7.6	82.3	7.9	0.306
	Post	71.5	7.9	81.8	7.7	92.0	9.3	
(3) Eating duration	Pre	66.4	11.2	26.5	9	24.7	10.6	0.812
	Post	72.6	10.6	25	10.2	24.7	15.4	
(4) Frequency of symptoms	Pre	63.5	3.6	72.4	3.7	72.2	4.1	0.029^*∗*^
	Post	73.3	4.3	71.6	4	75.7	5.6	
(5) Food selection	Pre	74.2	7.8	87.6	3.9	85.5	5.1	0.539
	Post	86.8	8.7	89.1	4.5	81.3	5	
(6) Communication	Pre	47.3	4.4	45.6	6.2	45.3	5.5	0.713
	Post	57.9	7	50.3	6.1	57.8	6.9	
(7) Fear to eat	Pre	72.5	5.3	71.6	4.6	73.2	5.4	0.662
	Post	80.9	6.6	73.9	5	74.3	5.9	
(8) Mental health	Pre	74	6.7	87.8	7.6	92.1	6.2	0.004^*∗*^
	Post	70.9	9.6	90.3	5.7	82.9	12.3	
(9) Social function	Pre	76.1	5.7	86.7	5.1	88.8	4.7	0.425
	Post	83.4	7.1	88.6	4.6	80.5	8.6	
(10) Sleep	Pre	50.9	11.4	15.5	11	25.1	10.2	0.093
	Post	65.9	11.4	26.4	10.2	18.9	11.2	
(11) Fatigue	Pre	65.7	7.5	51.4	7.7	60.4	4.8	0.298
	Post	81.4	5	58.7	4.2	56.2	10	
Total	Pre	63.9	3.5	67.5	4.1	68.9	3.1	0.288
	Post	72.2	4.7	69.3	3.2	69.6	4.3	

Statistical test: GEE; ^*∗*^*p* < 0.005; SWAL QOL: Quality of Life in Swallowing Disorders Questionnaire.
